# Incidence and relative risk of stroke in the diabetic and the non-diabetic population between 1998 and 2014: A community-based stroke register

**DOI:** 10.1371/journal.pone.0188306

**Published:** 2017-11-16

**Authors:** Andrea Icks, Heiner Claessen, Tatjana Kvitkina, Maria Narres, Michael Weingärtner, Stefan Schwab, Peter L. Kolominsky-Rabas

**Affiliations:** 1 Institute of Health Services Research and Health Economics, Centre for Health and Society, Heinrich Heine University, Düsseldorf, Germany; 2 Institute for Health Services Research and Health Economics, German Diabetes Center at the Heinrich Heine University Düsseldorf, Leibniz Institute for Diabetes Research, Düsseldorf, Germany; 3 German Centre for Diabetes Research, Neuherberg, Germany; 4 Interdisciplinary Centre for Health Technology Assessment (HTA) and Public Health, Friedrich Alexander University of Erlangen-Nürnberg, Erlangen, Germany; 5 National Information System of Federal Health Monitoring (Gesundheitsberichterstattung des Bundes), Erlangen Stroke Project (ESPro), Erlangen, Germany; 6 Department of Neurology, University Hospital Erlangen, Erlangen, Germany; Medizinische Universitat Innsbruck, AUSTRIA

## Abstract

One major objective of the St. Vincent Declaration was to reduce excess risk of stroke in people with diabetes mellitus. The aim of this study is to estimate the trend of incidence and relative risk of stroke in the diabetic and the non-diabetic populations in Germany over a 17-year period. We estimated age–sex standardised incidence rates of all stroke and ischaemic stroke in people with and without diabetes based on an ongoing prospective community-based stroke register covering 105,000 inhabitants. Time trends were analysed using Poisson regression. In total, 3,111 individuals (diabetes: 28.4%, men 46.9%, mean age 73.1 years (SD 13.2)) had a first stroke, 84.9% of which were ischaemic stroke. Among people with diabetes we observed a significant reduction in all stroke incidence by 1.5% per year (relative risk: 0.985; 95% confidence interval 0.972–0.9995) Likewise, this incidence tended to decrease for ischaemic stroke by 1% per year (0.993; 0.979–1.008). In contrast, the incidence rate for all stroke remained nearly stable among people without diabetes (1.003; 0.993–1.013) and for ischaemic stroke (1.002; 0.991–1.013). The relative risk comparing diabetic and non-diabetic population decreased for all stroke (two percent annual reduction) but not for ischaemic stroke. Time trends were similar for both sexes regarding all and ischaemic strokes. We found a reduction in risk of stroke in the diabetic population while this rate did not materially change in the non-diabetic population.

## Introduction

One of the primary objectives of the St. Vincent declaration was the decrease of stroke risk among persons with diabetes towards to that risk of the non-diabetic population [1, 2]. Several studies found an about two- to threefold elevated risk of stroke among individuals with diabetes compared to subjects without diabetes with particular high relative risks (RR) in the younger population [3–7].

However, only few studies evaluated the incidence rate (IR) of stroke in the diabetic compared with the non-diabetic population and their RRs. In a previous study analysing the IR of stroke in health insurance data for the years 2005–2007 in Germany [8], we found the IR of stroke in the diabetic population to be approximately double that in the non-diabetic population. However, no investigation of time trend could be considered due to the short time span.

Several studies from Western Europe and the USA indicate that the IR of stroke is declining [9–12]. However, it is unknown whether the decline has also been observed in people with diabetes or whether the gap between the diabetic and non-diabetic populations has narrowed. We found only two studies analysing trends of the IR of stroke in the diabetic compared with the non-diabetic population: Rautio and colleagues found declining IR of stroke in Sweden in non-diabetic men and women and diabetic women, but not in men with diabetes [13]. In Spain, Muñoz and colleagues found stable IR of haemorrhagic stroke in the diabetic population, whereas it decreased substantially in the non-diabetic population [14].

The objective of this study was to estimate the IR of stroke in the diabetic and the non-diabetic population as well as the RR and the investigation of time trends over a period of 17 years (1998–2014).

## Materials and methods

### Study population and data assessment

We analysed data from the Erlangen Stroke Project (ESPro), which is an ongoing prospective community-based stroke register in Germany covering a total population of 105,000 inhabitants. Since 1994, ESPro has been monitoring IR, risk factors, aetiology, and long-term outcome of stroke [15]. The characteristics of the study population, investigations and methods of assessment have been described in detail elsewhere [12, 16]. In brief, a number of sources with particularly overlapping information was applied to ensure complete case ascertainment as suggested as international gold-standard approach by Feigin et al. [17]. (1) hospital admission, computer-linked records systems and discharge lists; (2) regular checks of all relevant residential and hospital wards and nursing homes; (3) records of ambulance and emergency services; (4) death certificates and (5) general practitioners [16]. For the present study we included all hospitalised and non-hospitalised patients with suspected fatal or non-fatal stroke between 1 January 1998 and 31 December 2014.

A study clinician defined stroke diagnosis according to the criteria of the World Health Organization [18] and imaging. Patients with first-ever stroke (ischaemic stroke, intracerebral haemorrhage, subarachnoid haemorrhage, and stroke of uncertain cause) were included in the present study [19]. Persons with transient ischaemic attacks (TIA) were only registered but not further assessed and therefore excluded [16], since the WHO definition on stroke does not meet the criteria for TIA. We assessed IR of stroke (ischaemic strokes as subgroup analysis) with regard to age, sex, diabetes status, and date of the first stroke. Furthermore, we also described all incident cases with regard to smoking status, socioeconomic status (education), and comorbidities (myocardial infarction and hypertension).

A person was classified as having diabetes by 1) use of anti-hyperglycaemic drugs, 2) a fasting blood glucose level of 126 mg/dl and HbA1c >6.5 or above, and 3) self-report of physician-diagnosed diagnosis. The latter information was verified by checking the records of general practitioners and care protocols.

Data of the population of Erlangen were obtained from the Federal Office for Statistics [20]. The diabetic population was estimated in the adult population based on age- (18–39, 40–49, 50–59, 60–69, 70–79, and 80+ years) and sex-specific diabetes prevalence from two German nationwide surveys (German Health Interview and Examination Surveys (GNHIES98), DEGS1)) [21–23] conducted in 1997–99 and 2008–11 respectively. Diabetes was defined in both surveys based on self-report of physician-diagnosed diabetes, intake of antihyperglycaemic medication as well as an HbA1c value ≥ 6.5% within the last week before the survey [24]. The Robert Koch Institute regularly conducts health interview surveys as a part of its nationwide health monitoring. These two surveys are the only nationwide data sources with a comparable study design to estimate reliable age–sex specific diabetes prevalence covering more than a decade. As both surveys only covered the age range 18–79 years, we assumed that the estimated diabetes prevalence remained constant in the oldest age group (80+ years), which has been previously shown to be reliable [21].

We assumed that the estimated age- and sex-specific diabetes prevalence linearly increased from 1998 to 2011 using the estimators of GNHIES98 for the first and DEGS1 for the last year. We further assumed that the estimated diabetes prevalence continued to rise up to 2014 with the same increase.

### Statistical analyses

The main analyses were conducted for the entire population as well as separately for men and women. We computed stratum-specific and age-sex standardised IRs of stroke with 95% CIs in the estimated population with and without diabetes for each calendar year using the German population of 2005 as standard population. The number of persons with diabetes was estimated by multiplying the estimated diabetes prevalence of each age and sex stratum with the corresponding study population of Erlangen. We calculated person years by taking the estimated number of persons with and without diabetes for each calendar year. Furthermore, we estimated RRs comparing diabetic versus non-diabetic populations from the standardized IRs.

In order to investigate time trends, we first performed separate Poisson regression models with IRs of all stroke as dependent variable for individuals with as well as without diabetes using year of stroke diagnosis as linear continuous difference from baseline year 1998 and age as independent variables. The two lowest age classes (i.e. 18–39, 40–49 years) were combined to one group (18–49 years) due to convergence problems of some models and were therefore used as reference group. Furthermore, we fitted analogous Poisson models to the entire population. In these models, we additionally included a variable presence of diabetes (yes vs. no) as well as an interaction term for diabetes and years since 1998.

In a sensitivity analysis, the main analyses were repeated assuming that the estimated age- and sex-specific diabetes prevalence remained constant for the years 2011–2014 since it was discussed whether the estimated diabetes prevalence remained constant or further increased in the later years. In order to take into account a potential misclassification bias due to first-ever all strokes with an unknown diabetes status, we further computed the main analyses, counting all these cases first as diabetic and second as non-diabetic.

The main analyses were also repeated for strokes due to ischaemic stroke.

To take into account over-dispersion of the outcome variable, all analyses were conducted with the de-scale adjustment [25], which was based on cumulated data on the covariate strata year*sex*age class*diabetes. We performed analysis using the Statistical Analysis System SAS (SAS for Windows 7, Release 9.4 TS1M1, SAS Institute Inc. Cary, NC, USA).

ESPro was approved by the local ethics committee. Patients or their legal representatives gave their written informed consent for participation.

## Results

### Study population

The description of the study population is presented in Table 1. The data covered the adult (≥ 18 years) population of Erlangen (1998: 83,584, 2014: 90,428). Diabetes prevalence in the Erlangen population increased from 5.6% in 1998 to 8.2% in 2014, with higher values in the female population.

**Table 1:** Description of persons with first stroke, and the background population, Erlangen, 1998–2014.

						men		women	
	total	men	women	diabetes	no diabetes	diabetes**	no diabetes**	diabetes**	no diabetes**
number of persons with first stroke (%*)	3,111 (100.0)	1,458 (46.9)	1,653 (53.1)	884 (28.4)	2,227 (71.6)	435 (29.8)	1,023 (70.2)	449 (27.2)	1,204 (72.8)
Person years (%)	1,481,658(100.0)	718,669 (48.5)	762,989 (51.5)	101,456 (6.8)	1,380,202 (93.2)	43,641 (6.1)	675,028 (93.9)	57,815 (7.6)	705,174 (92.4)
Mean age*** (years, SD)	73.1 (13.2)	70.1 (12.5)	75.8 (13.3)	74.9 (10.6)	72.4 (14.0)	71.4 (10.3)	69.5 (13.3)	78.3 (9.8)	74.9 (14.2)
Age class									
18–39 (%)	60 (1.9)	23 (1.6)	37 (2.2)	1 (0.1)	59 (2.6)	0 (0.0)	23 (2.2)	1 (0.2)	36 (3.0)
40–49 (%)	140 (4.5)	77 (5.3)	63 (3.8)	19 (2.1)	121 (5.4)	14 (3.2)	63 (6.2)	5 (1.1)	58 (4.8)
50–59 (%)	292 (9.4)	196 (13.4)	96 (5.8)	65 (7.4)	227 (10.2)	47 (10.8)	149 (14.6)	18 (4.0)	78 (6.5)
60–69 (%)	571 (18.4)	352 (24.1)	219 (13.2)	171 (19.3)	400 (18.0)	120 (27.6)	232 (22.7)	51 (11.4)	168 (14.0)
70–79 (%)	973 (31.3)	474 (32.5)	499 (30.2)	322 (36.4)	651 (29.2)	161 (37.0)	313 (30.6)	161 (35.9)	338 (28.1)
≥ 80 (%)	1,075 (34.6)	336 (23.0)	739 (44.7)	306 (34.6)	769 (34.5)	93 (21.4)	243 (23.8)	213 (47.4)	526 (43.7)
Smoking status									
Smoker (%)	542 (17.4)	329 (22.6)	213 (12.9)	146 (16.5)	396 (17.8)	101 (23.2)	228 (22.3)	45 (10.0)	168 (14.0)
Ex-smoker (%)	612 (19.7)	469 (32.2)	143 (8.7)	179 (20.2)	433 (19.4)	145 (33.3)	324 (31.7)	34 (7.6)	109 (9.1)
Non-smoker (%)	767 (24.7)	237 (16.3)	530 (32.1)	191 (21.6)	576 (25.9)	64 (14.7)	173 (16.9)	127 (28.3)	403 (33.5)
Unknown (%)	1,190 (38.3)	423 (29.0)	767 (46.4)	368 (41.6)	822 (36.9)	125 (28.7)	298 (29.1)	243 (54.1)	524 (43.5)
Highest degree of education									
No graduation (%)	60 (1.9)	27 (1.9)	33 (2.0)	16 (1.8)	44 (2.0)	7 (1.6)	20 (2.0)	9 (2.0)	24 (2.0)
Primary school (%)	1,173 (37.7)	497 (34.1)	676 (40.9)	357 (40.4)	816 (36.6)	160 (36.8)	337 (32.9)	197 (43.9)	479 (39.8)
Secondary school (%)	456 (14.7)	214 (14.7)	242 (14.6)	114 (12.9)	342 (15.4)	64 (14.7)	150 (14.7)	50 (11.1)	192 (15.9)
Baccalaureate (%)	172 (5.5)	89 (6.1)	83 (5.0)	36 (4.1)	136 (6.1)	26 (6.0)	63 (6.2)	10 (2.2)	73 (6.1)
University (%)	365 (11.7)	295 (20.2)	70 (4.2)	76 (8.6)	289 (13.0)	67 (15.4)	228 (22.3)	9 (2.0)	61 (5.1)
Unknown (%)	885 (28.4)	336 (23.0)	549 (33.2)	285 (32.2)	600 (26.9)	111 (25.5)	225 (22.0)	174 (38.8)	375 (31.1)
Diagnosis of myocardial infarction									
Yes (%)	289 (9.3)	177 (12.1)	112 (6.8)	102 (11.5)	187 (8.4)	69 (15.9)	108 (10.6)	33 (7.3)	79 (6.6)
No (%)	2,499 (80.3)	1,148 (78.7)	1,351 (81.7)	666 (75.3)	1,833 (82.3)	319 (73.3)	829 (81.0)	347 (77.3)	1,004 (83.4)
Unknown (%)	323 (10.4)	133 (9.1)	190 (11.5)	116 (13.1)	207 (9.3)	47 (10.8)	86 (8.4)	69 (15.4)	121 (10.0)
Diagnosis of hypertension									
Yes (%)	2,357 (75.8)	1,085, (74.4)	1,272, (77)	777 (87.9)	1,580 (70.9)	376 (86.4)	709 (69.3)	401 (89.3)	871 (72.3)
No (%)	109 (3.5)	54, (3.7)	55 (3.3)	22 (2.5)	87 (3.9)	11 (2.5)	43 (4.2)	11 (2.4)	44 (3.7)
Unknown (%)	645 (20.7)	319, (21.9)	326 (19.7)	85 (9.6)	560 (25.1)	48 (11.0)	271 (26.5)	37 (8.2)	289 (24.0)
Number of first strokes by type									
Ischaemic stroke (%)	2,640 (84.9)	1,240 (85)	1,400 (84.7)	775 (87.7)	1,865 (83.7)	379 (87.1)	861 (84.2)	396 (88.2)	1,004 (83.4)
Intracerebral haemorrhage (%)	357 (11.5)	178 (12.2)	179 (10.8)	87 (9.8)	270 (12.1)	48 (11.0)	130 (12.7)	39 (8.7)	140 (11.6)
Subarachnoid haemorrhage (%)	85 (2.7)	31 (2.1)	54 (3.3)	9 (1.0)	76 (3.4)	4 (0.9)	27 (2.6)	5 (1.1)	49 (4.1)
Stroke of uncertain cause (%)	29 (0.9)	9 (0.6)	20 (1.2)	13 (1.5)	16 (0.7)	4 (0.9)	5 (0.5)	9 (2.0)	11 (0.9)
Number of first strokes per year									
1998 (%*)	179 (100.0)	72 (40.2)	107 (59.8)	57 (31.8)	122 (68.2)	27 (37.5)	45 (62.5)	30 (28.0)	77 (72.0)
1999 (%*)	192 (100.0)	97 (50.5)	95 (49.5)	62 (32.3)	130 (67.7)	34 (35.1)	63 (64.9)	28 (29.5)	67 (70.5)
2000 (%*)	172 (100.0)	81 (47.1)	91 (52.9)	50 (29.1)	122 (70.9)	19 (23.5)	62 (76.5)	31 (34.1)	60 (65.9)
2001 (%*)	141 (100.0)	64 (45.4)	77 (54.6)	53 (37.6)	88 (62.4)	22 (34.4)	42 (65.6)	31 (40.3)	46 (59.7)
2002 (%*)	141 (100.0)	76 (53.9)	65 (46.1)	43 (30.5)	98 (69.5)	21 (27.6)	55 (72.4)	22 (33.8)	43 (66.2)
2003 (%*)	158 (100.0)	63 (39.9)	95 (60.1)	44 (27.8)	114 (72.2)	20 (31.7)	43 (68.3)	24 (25.3)	71 (74.7)
2004 (%*)	193 (100.0)	80 (41.5)	113 (58.6)	44 (22.8)	149 (77.2)	21 (26.3)	59 (73.8)	23 (20.4)	90 (79.6)
2005 (%*)	216 (100.0)	101 (46.8)	115 (53.2)	55 (25.5)	161 (74.5)	27 (26.7)	74 (73.3)	28 (24.3)	87 (75.7)
2006 (%*)	181 (100.0)	81 (44.8)	100 (55.2)	51 (28.2)	130 (71.8)	19 (23.5)	62 (76.5)	32 (32.0)	68 (68.0)
2007 (%*)	169 (100.0)	89 (52.7)	80 (47.3)	41 (24.3)	128 (75.7)	24 (27.0)	65 (73.0)	17 (21.3)	63 (78.8)
2008 (%*)	176 (100.0)	92 (52.3)	84 (47.7)	52 (29.5)	124 (70.5)	28 (30.4)	64 (69.6)	24 (28.6)	60 (71.4)
2009 (%*)	166 (100.0)	68 (41.0)	98 (59.0)	49 (29.5)	117 (70.5)	23 (33.8)	45 (66.2)	26 (26.5)	72 (73.5)
2010 (%*)	192 (100.0)	86 (44.8)	106 (55.2)	58 (30.2)	134 (69.8)	22 (25.6)	64 (74.4)	36 (34.0)	70 (66.0)
2011 (%*)	199 (100.0)	89 (44.7)	110 (55.3)	63 (31.7)	136 (68.3)	34 (38.2)	55 (61.8)	29 (26.4)	81 (73.6)
2012 (%*)	193 (100.0)	98 (50.8)	95 (49.2)	51 (26.4)	142 (73.6)	32 (32.7)	66 (67.3)	19 (20.0)	76 (80.0)
2013 (%*)	218 (100.0)	114 (52.3)	104 (47.7)	58 (26.6)	160 (73.4)	34 (29.8)	80 (70.2)	24 (23.1)	80 (76.9)
2014 (%*)	225 (100.0)	107 (47.6)	118 (52.4)	53 (23.6)	172 (76.4)	28 (26.2)	79 (73.8)	25 (21.2)	93 (78.8)
Number of person years per year									
1998 (%*)	83,584	40,183 (48.1)	43,401 (51.9)	4,643 (5.6)	78,941 (94.4)	1,887 (4.7)	38,296 (95.3)	2,756 (6.4)	40,645 (93.6)
1999 (%*)	83,760	40,309 (48.1)	43,451 (51.9)	4,798 (5.7)	78,962 (94.3)	1,958 (4.9)	38,351 (95.1)	2,840 (6.5)	40,611 (93.5)
2000 (%*)	83,932	40,383 (48.1)	43,549 (51.9)	4,969 (5.9)	78,963 (94.1)	2,035 (5.0)	38,348 (95.0)	2,934 (6.7)	40,615 (93.3)
2001 (%*)	84,980	40,993 (48.2)	43,987 (51.8)	5,150 (6.1)	79,830 (93.9)	2,126 (5.2)	38,867 (94.8)	3,024 (6.9)	40,963 (93.1)
2002 (%*)	85,198	41,086 (48.2)	44,112 (51.8)	5,277 (6.2)	79,921 (93.8)	2,199 (5.4)	38,887 (94.6)	3,078 (7.0)	41,034 (93.0)
2003 (%*)	85,436	41,259 (48.3)	44,177 (51.7)	5,413 (6.3)	80,023 (93.7)	2,278 (5.5)	38,981 (94.5)	3,135 (7.1)	41,042 (92.9)
2004 (%*)	85,704	41,355 (48.3)	44,349 (51.7)	5,591 (6.5)	80,113 (93.5)	2,370 (5.7)	38,985 (94.3)	3,221 (7.3)	41,128 (92.7)
2005 (%*)	86,222	41,711 (48.4)	44,511 (51.6)	5,732 (6.6)	80,490 (93.4)	2,448 (5.9)	39,263 (94.1)	3,284 (7.4)	41,227 (92.6)
2006 (%*)	86,905	42,038 (48.4)	44,867 (51.6)	5,911 (6.8)	80,994 (93.2)	2,540 (6.0)	39,498 (94.0)	3,371 (7.5)	41,496 (92.5)
2007 (%*)	87,830	42,584 (48.5)	45,246 (51.5)	6,108 (7.0)	81,722 (93.0)	2,641 (6.2)	39,943 (93.8)	3,467 (7.7)	41,779 (92.3)
2008 (%*)	88,182	42,858 (48.6)	45,324 (51.4)	6,279 (7.1)	81,903 (92.9)	2,730 (6.4)	40,128 (93.6)	3,549 (7.8)	41,775 (92.2)
2009 (%*)	88,745	43,128 (48.6)	45,617 (51.4)	6,464 (7.3)	82,281 (92.7)	2,822 (6.5)	40,306 (93.5)	3,642 (8.0)	41,975 (92.0)
2010 (%*)	88,978	43,239 (48.6)	45,739 (51.4)	6,645 (7.5)	82,333 (92.5)	2,925 (6.8)	40,314 (93.2)	3,720 (8.1)	42,019 (91.9)
2011 (%*)	90,888	44,506 (49.0)	46,382 (51.0)	6,873 (7.6)	84,015 (92.4)	3,042 (6.8)	41,464 (93.2)	3,831 (8.3)	42,551 (91.7)
2012 (%*)	90,307	44,231 (49.0)	46,076 (51.0)	7,013 (7.8)	83,294 (92.2)	3,111 (7.0)	41,120 (93.0)	3,902 (8.5)	42,174 (91.5)
2013 (%*)	90,579	44,423 (49.0)	46,156 (51.0)	7,210 (8.0)	83,369 (92.0)	3,220 (7.2)	41,203 (92.8)	3,990 (8.6)	42,166 (91.4)
2014 (%*)	90,428	44,383 (49.1)	46,045 (50.9)	7,380 (8.2)	83,048 (91.8)	3,309 (7.5)	41,074 (92.5)	4,071 (8.8)	41,974 (91.2)

* Percentages related to all persons with first stroke.

** Percentages related to total male population and female population, respectively.

*** Age at time of first stroke.

In total, we identified 3,579 people with a first-ever stroke in the years 1998–2014. We excluded 468 individuals, as their diabetes status was not known. Of the remaining 3,111, 28.4% were classified as having diabetes (antihyperglycaemic drugs 74.6%; self-reports of physician-diagnosed diagnosis 15.6% and laboratory findings 9.7%). 53.1% were female with no consistent change in proportion over time. The mean age at the time of first-ever stroke was 73.1 years (standard deviation (SD) 13.2), which remained nearly stable over the time period, with higher values in women (75.8 years, SD 13.3) and individuals with diabetes (74.9 years, SD 10.6). At 84.9% ischaemic stroke was the most common stroke type followed by intercerebral haemorrhage (11.5%) and subarachnoidal haemorrhage (2.7%). These proportions remained nearly stable over the study period and were comparable among all subgroups.

### Incidence rates and relative risks

**Fig 1:**
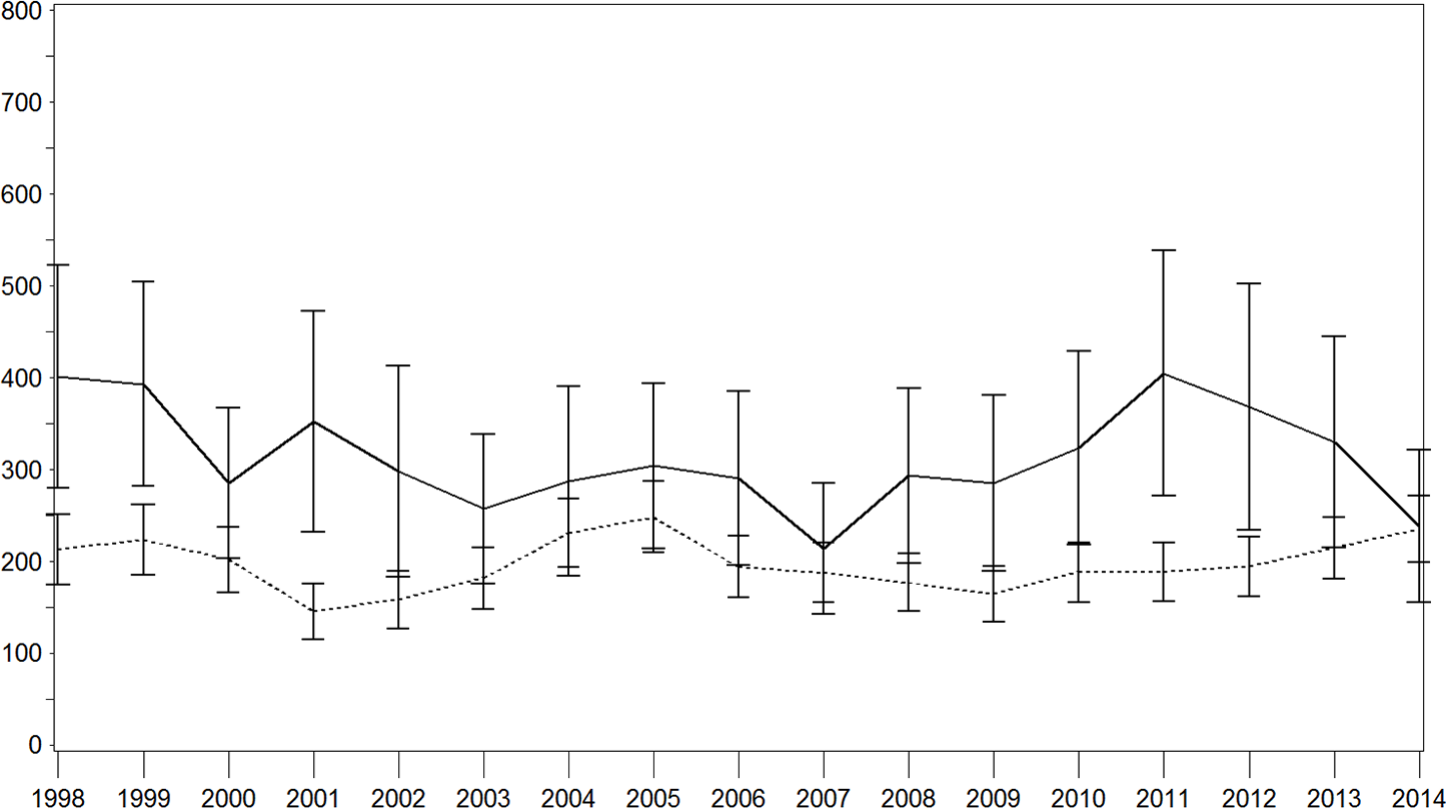
Age–sex standardised incidence rate of stroke with and without diabetes in the total population. Continuous lines = persons with diabetes; dotted lines = persons without diabetes; x-axis: calendar year; y-axis: incidence rate per 100,000 person years.

**Fig 2:**
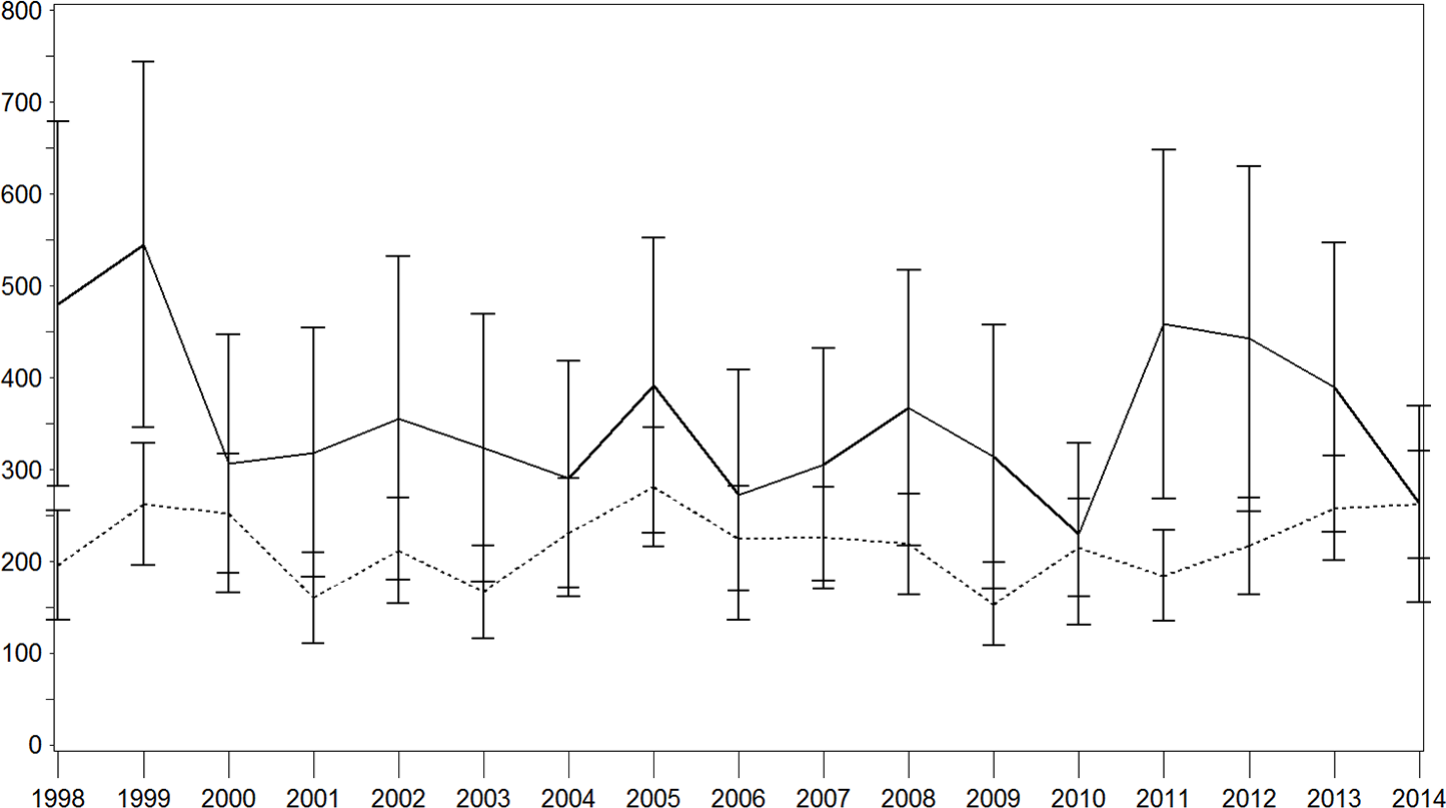
Age–sex standardised incidence rate of stroke with and without diabetes in the male population. Continuous lines = persons with diabetes; dotted lines = persons without diabetes; x-axis: calendar year; y-axis: incidence rate per 100,000 person years.

**Fig 3:**
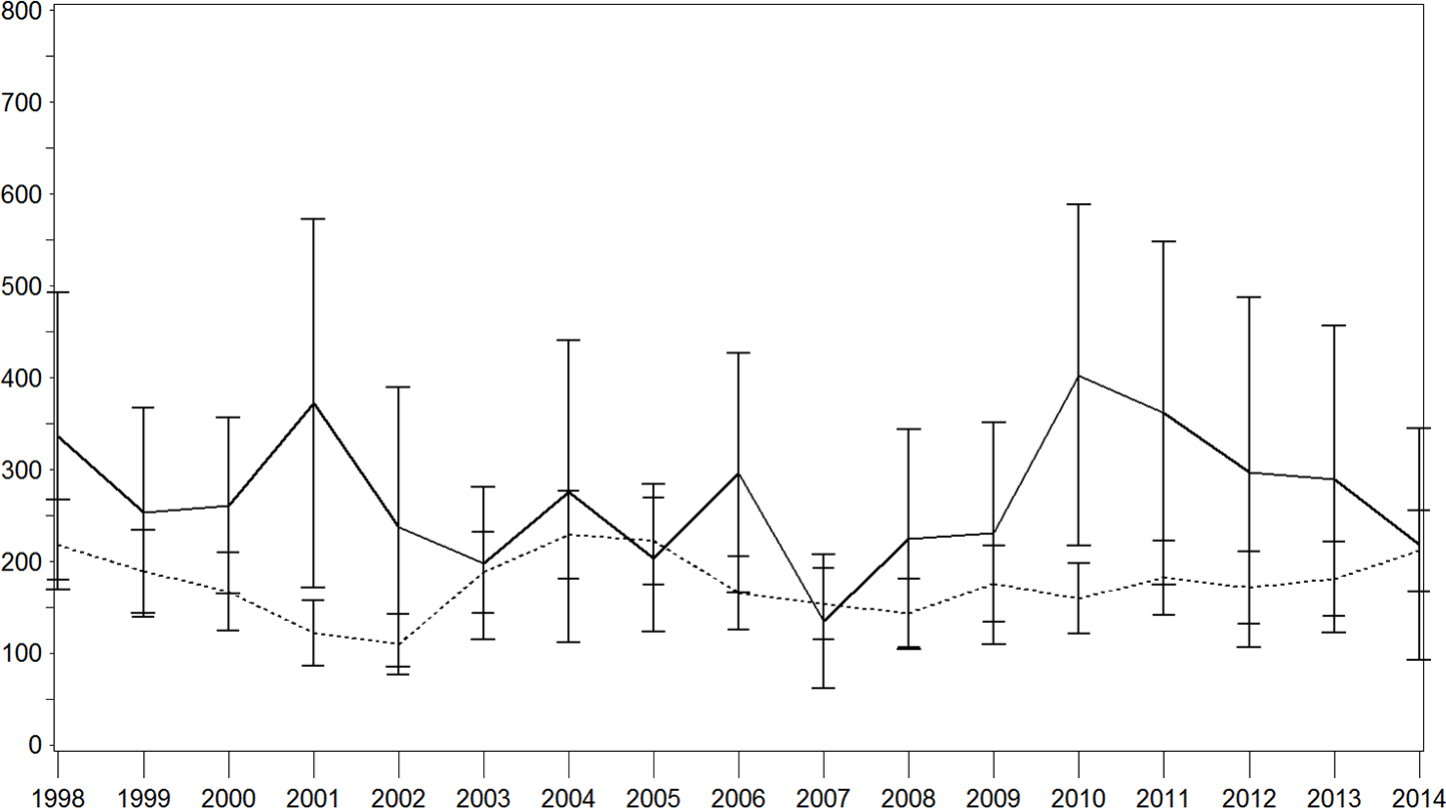
Age–sex standardised incidence rate of stroke with and without diabetes in the female population. Continuous lines = persons with diabetes; dotted lines = persons without diabetes; x-axis: calendar year; y-axis: incidence rate per 100,000 person years.

Age–sex standardized IRs of all stroke for each year are shown in Figs 1–3. There were brief variations, which was particularly true in the population with diabetes (Fig 1). Over the whole study period, we observed a decrease in the IR per 100,000 person years in the population with diabetes (1998: 401.2 [95% confidence interval (CI) 279.4–523.1]; 2014: 238.5 [155.8–321.2]) with a particularly strong decrease in the last 3 years. This pattern was similar for both sexes with higher IRs in the male-population (Figs 2 and 3). In contrast, this IR remained nearly constant in the population without diabetes, with a moderate increase in the last 3 years (1998: 212.6 [174.5–250.6]; 2014: 235.2 [199.2–271.2]). With regard to the population with diabetes, higher IR were seen among men while these results were comparable for both sexes in the population without diabetes.

The RR of all stroke in the population with diabetes compared with the population without diabetes decreased over the whole study period and was highest in 2001 (RR: 2.4 [95% CI 1.6–3.6]) and lowest with no difference in 2014 (RR: 1.0 [0.7–1.5]). Th RR was somewhat higher among men in the first years of the study period while similar values were seen for the later years in both sexes (data not shown).

**Fig 4:**
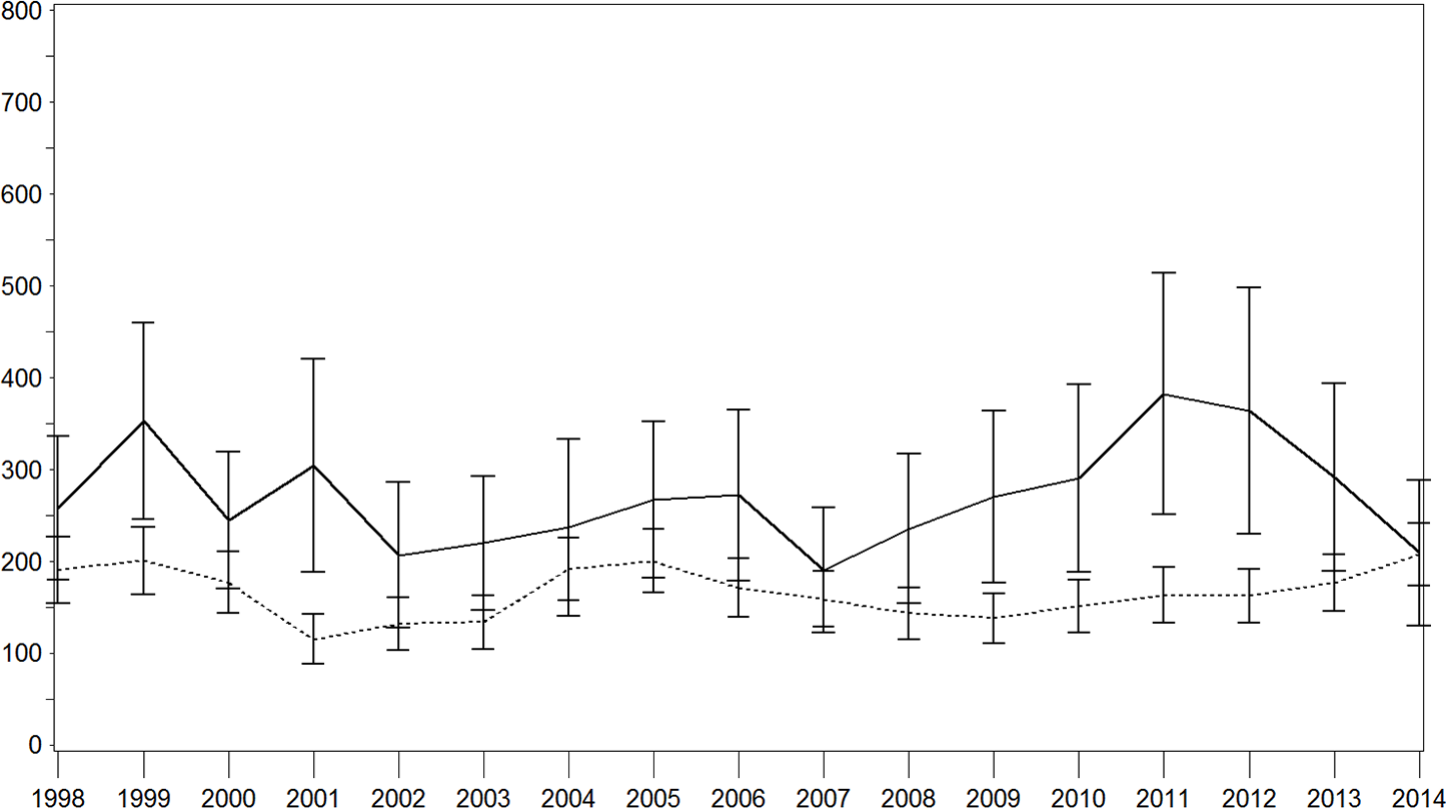
Age–sex standardised incidence rate of ischaemic stroke with and without diabetes in the total population. Continuous lines = persons with diabetes; dotted lines = persons without diabetes; x-axis: calendar year; y-axis: incidence rate per 100,000 person years.

**Fig 5:**
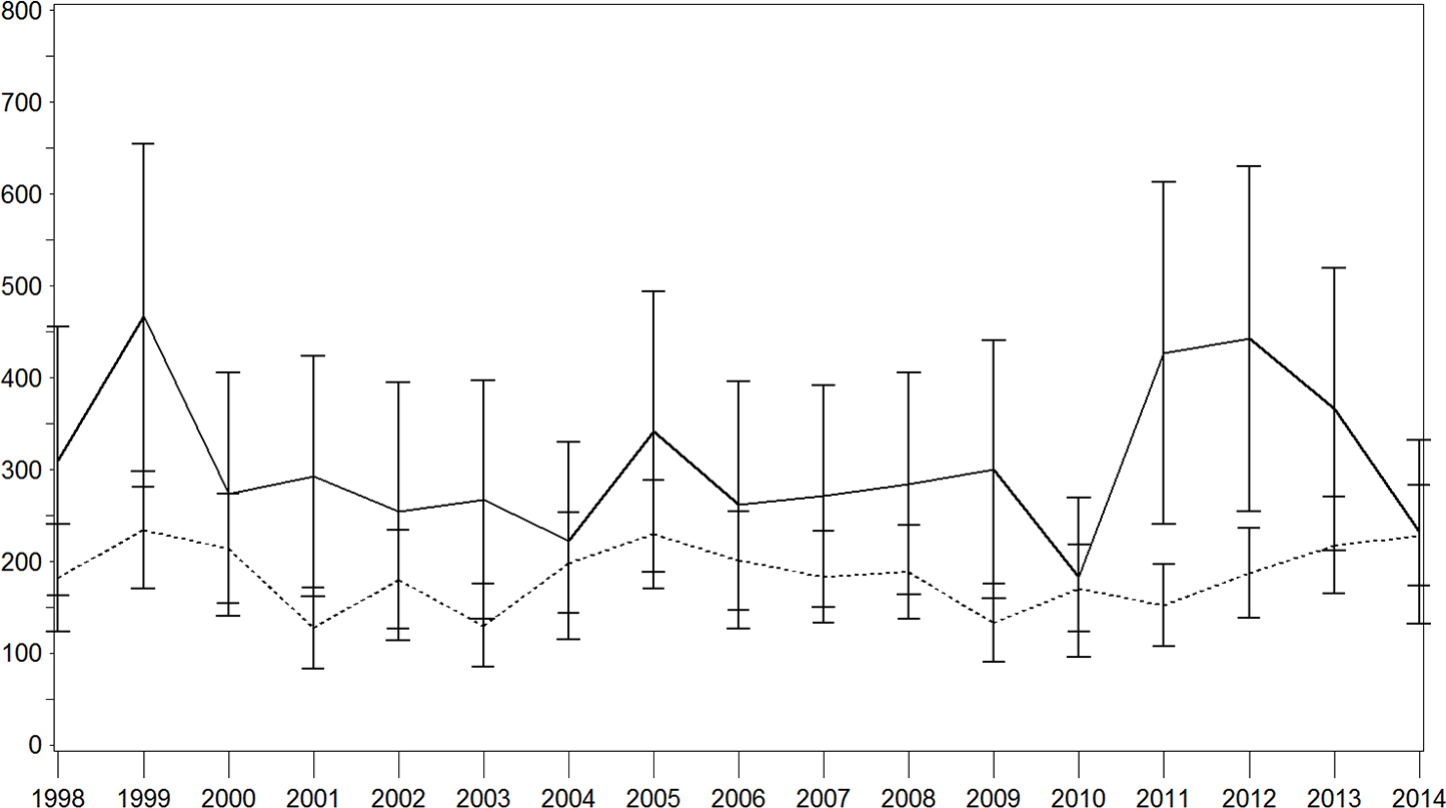
Age–sex standardised incidence rate of ischaemic stroke with and without diabetes in the male population. Continuous lines = persons with diabetes; dotted lines = persons without diabetes; x-axis: calendar year; y-axis: incidence rate per 100,000 person years.

**Fig 6:**
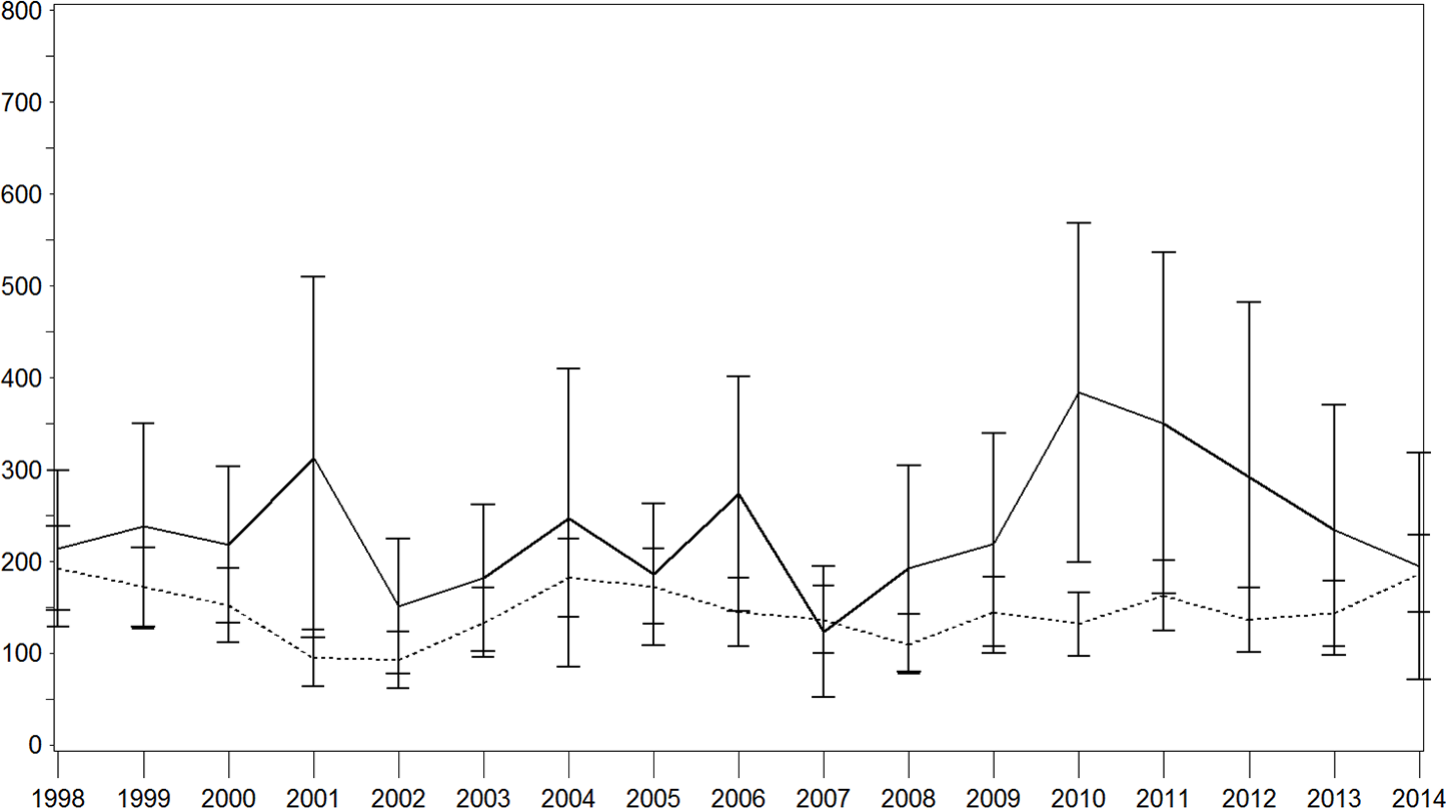
Age–sex standardised incidence rate of ischaemic stroke with and without diabetes in the female population. Continuous lines = persons with diabetes; dotted lines = persons without diabetes; x-axis: calendar year; y-axis: incidence rate per 100,000 person years.

When repeating the analyses for ischaemic stroke (Figs 4–6), we observed only a slight decrease in IR per 100,000 person years in the population with diabetes (1998: 258.1 [179.7–336.4]; 2014: 209.4 [130.0–288.9]), while this IR remained nearly constant in the population without diabetes (1998: 190.4 [154.3–226.6]; 2014: 207.6 [173.8–241.5]). The RR ranged between 2.6 [1.7–4.1] in 2001 and 1.0 [0.7–1.5] in 2014. When stratifying for sex, we observed similar results despite increased variation of IRs, with higher IRs for men in the diabetic population.

### Analysis of time trend

Table 2 shows results of the incidence trend from the fully adjusted Poisson models. The RRs in the population with and without diabetes are shown in models 1a and 1b. During the observation period we observed a significant decrease of all stroke incidence, by one and a half percent per year (RR per calendar year: 95% CI: 0.985; 0.972–0.9995), in the population with diabetes, with similar results among men and women with the exception that these trends were not significant due to a smaller sample size. In contrast, the trend of incidence remained constant among individuals without diabetes (RR per calendar year 1.003; 0.993–1.013), which was true for both sexes. When considering the entire population in model 2 we observed a significant increased IR in the population with diabetes compared with the population without diabetes. This difference was particularly strong among the younger age groups but was not significant in the oldest age group (RR diabetes vs. no diabetes < 50 years: 3.43; 2.09–5.61; 80+ years: 1.11; 0.98–1.27) (data not shown). The interaction diabetes*calendar year was significant, indicating that this RR decreased by two percent per year (RR per calendar year 0.979; 0.960–0.997) with similar results in both sexes (model 2). These results did not alter when assuming a constant estimated diabetes prevalence from 2011 (S1 Fig, S1 Table). Likewise, the results regarding time trend did not materially change when counting all strokes with unknown diabetes first as diabetic (S2 Fig, S2 Table) and second as non-diabetic cases (S3 Fig, S3 Table).

**Table 2:** Results of Poisson models*: Relative risks for Stroke, Erlangen, 1998–2014.

variables	relative risk for stroke (95% CI)		
All strokes	total population	men	women
Model 1a (persons with diabetes)			
Calendar year	0.985 (0.972–0.9995)**	0.987 (0.968–1.007)	0.985 (0.967–1.004)
Male vs. female	1.254 (1.089–1.443)**	---------	---------
Age (years)***			
≥ 80	17.963 (11.209–28.787)**	9.847 (5.620–17.254)**	34.681 (15.436–77.920)**
70–79	10.976 (6.856–17.573)**	7.907 (4.586–13.631)**	18.118 (8.034–40.857)**
60–69	6.600 (4.071–10.701)**	5.348 (3.080–9.288)**	8.509 (3.657–19.803)**
50–59	4.410 (2.613–7.443)**	3.084 (1.701–5.591)**	7.528 (2.994–18.929)**
Model 1b (persons without diabetes)			
Calendar year	1.003 (0.993–1.013)	1.003 (0.991–1.016)	1.003 (0.989–1.017)
Male vs. female	1.233 (1.117–1.361)**	---------	---------
Age (years)***			
≥ 80	56.831 (46.908–68.853)**	56.607 (44.655–71.758)**	51.784 (39.893–67.218)**
70–79	28.096 (23.134–34.121)**	33.575 (26.680–42.252)**	23.170 (17.660–30.399)**
60–69	12.637 (10.274–15.542)**	17.187 (13.542–21.812)**	8.918 (6.607–12.037)**
50–59	5.462 (4.340–6.875)**	7.947 (6.154–10.263)**	3.35 (2.345–4.786)**
Model 2			
Calendar year	1.004 (0.994–1.014)	1.005 (0.991–1.018)	1.003 (0.990–1.017)
Diabetes (yes vs. no)	1.728 (1.442–2.065)**	1.890 (1.480–2.401)**	1.558 (1.227–1.969)**
Male vs. female	1.250 (1.149–1.359)**	---------	---------
Age (years)***			
≥ 80	49.507 (41.477–59.503)**	44.855 (35.318–57.543)**	48.946 (38.780–62.644)**
70–79	26.091 (21.840–31.382)**	29.189 (23.190–37.158)**	22.956 (18.059–29.561)**
60–69	12.729 (10.552–15.442)**	16.342 (12.903–20.914)**	9.205 (7.062–12.106)**
50–59	5.776 (4.680–7.148)**	7.738 (5.987–10.073)**	3.768 (2.739–5.179)**
Diabetes x calendar year	0.979 (0.960–0.997)**	0.977 (0.953–1.001)	0.981 (0.956–1.006)
** Ischaemic strokes only**	total population	men	women
Model 1a (persons with diabetes)			
Calendar year	0.993 (0.979–1.008)	0.999 (0.978–1.020)	0.990 (0.971–1.010)
Male vs. female	1.235 (1.068–1.426)**	---------	---------
Age (years)***			
≥ 80	20.034 (12.099–33.173) **	11.671 (6.258–21.767)**	36.452 (15.489–85.786)**
70–79	11.935 (7.212–19.752) **	8.481 (4.607–15.614)**	19.398 (8.214–45.809)**
60–69	7.269 (4.338–12.181) **	5.764 (3.106–10.696)**	9.701 (3.991–23.579)**
50–59	4.801 (2.751–8.378) **	3.527 (1.824–6.819)**	7.072 (2.643–18.922)**
Model 1b (persons without diabetes)			
Calendar year	1.002 (0.991–1.013)	1.003 (0.990–1.017)	1.002 (0.986–1.017)
Male vs. female	1.260 (1.132–1.403)**	---------	---------
Age (years)***			
≥ 80	71.647 (57.228–89.699) **	66.994 (50.982–88.035)**	69.234 (50.261–95.369)**
70–79	35.007 (27.892–43.937) **	38.631 (29.595–50.426)**	31.070 (22.328–43.236)**
60–69	15.031 (11.810–19.129) **	19.411 (14.732–25.577)**	11.041 (7.686–15.862)**
50–59	6.174 (4.723–8.070) **	8.601 (6.395–11.569)**	3.840 (2.496–5.908)**
Model 2			
Calendar year	1.003 (0.992–1.014)	1.005 (0.990–1.019)	1.002 (0.988–1.017)
Diabetes (yes vs. no)	1.655 (1.364–2.001)**	1.751 (1.341–2.273)**	1.533 (1.186–1.971)**
Male vs. female	1.266 (1.157–1.385)**	---------	---------
Age (years)***			
≥ 80	61.328 (50.088–75.843)**	53.227 (40.744–70.529)**	63.936 (48.429–86.265)**
70–79	31.829 (25.969–39.397)**	33.003 (25.461–43.446)**	30.234 (22.748–41.020)**
60–69	15.105 (12.197–18.864)**	18.294 (14.017–24.219)**	11.614 (8.512–16.099)**
50–59	6.559 (5.163–8.372)**	8.521 (6.386–11.489)**	4.281 (2.937–6.252)**
Diabetes x calendar year	0.987 (0.967–1.006)	0.988 (0.962–1.015)	0.986 (0.960–1.013)

*Models were adjusted for all variables included in this table.

**p-value < 0.05.

***Baseline: 18–49 years.

For ischaemic stroke, we observed a slight but no significant decrease in IR in the population with diabetes by one percent per year, (RR per calendar year 0.993; 0.979–1.008) with comparable results among men and women. Likewise, this IR remained nearly constant among people without diabetes (1.003; 0.992–1.014), with similar results for both sexes. The interaction diabetes*calendar year tended to decrease, however, was not significant, (0.987; 0.967–1.006), which was true for both sexes.

## Discussion

### Main findings

Our study is part of an evaluation of how well the St. Vincent objectives have been met in Germany. In our study region over the 17-year study period, we found a significant decrease in the IR of all stroke in the diabetic population. Considering solely ischaemic stroke in the diabetic population, the risk tended to decrease, however, not significantly, maybe due to low statistical power. No change was found in the non-diabetic population with regard to all and ischaemic stroke.

Our findings may indicate an improvement in diabetes care. Several health technologies have been introduced in the past decades, such as medication to reduce hypertension, one of the most important risk factors of stroke. National programmes designed to improve diabetes care, e.g. disease management programmes, have been implemented since the beginning of the 2000s. The National Health Surveys found substantially improvements between 1997 and 2011, e.g. regarding the proportions of people with diabetes achieving an HbA_1c_ <7.0% (32.4% vs 65.4%), a blood pressure <130/80 mmHg (32.0% vs. 47.2%.), total cholesterol <190 mg/dl (13.5% vs. 41.9%), with statin use (11.7% vs. 35.9%), eye (51.1% vs. 78.4%), and foot (48.0% vs. 61.4%) examination within past 12 months [26]. However, other explanations may be possible. For example, it may be that the characteristics of the background population changed due to migration. Furthermore, general stroke prevention interventions in Erlangen may have changed the stroke population.

### Comparison with other studies

We identified only two studies that analysed trends in the IR and RR of all stroke in the diabetic compared with the non-diabetic population. A Spanish study was restricted to haemorrhagic strokes [14]. Rautio and colleagues analysed all strokes and found declining stroke IRs in Sweden in non-diabetic men and women and diabetic women, but not in men with diabetes [13]. They did not find an explanation for this gender difference. Interestingly, in the Swedish region, the trend in the risk of myocardial infarction was also worse in diabetic men [13], and this was also observed in a German study [27]. In contrast, we did not find gender differences with regard to trend. Further studies are needed which look for gender differences in more detail.

### Strengths and limitations

A number of limitations have to be considered. First, the results of our study are dependent on the estimates of the number of diabetic individuals in the background population. We estimated diabetes prevalence using well-designed German health surveys, and performed sensitivity analyses, which resulted in stable estimates. In both surveys, diagnosed diabetes (GNHIES98, DEGS1) was uniformly defined by self-report of physician-diagnosed diagnosis, intake of antihyperglycaemic medication as well as an HbA1c value ≥ 6.5% within the last week before the survey [24]. Therefore, the definition of diabetes is not exactly the same as the case definition, however, quite similar. Our approach to estimate the background diabetic population using survey data has often been applied [28–30]. Unfortunately, we cannot exclude misclassification resulting in biased estimates of the IR and the RR. However, this was true for the whole observation period, hence, the time trend should not be affected. Second, we did not include clinical variables (e. g. population influx, changes of the provision of care considering stroke), since these data are missing for the background population. Third and last, we analysed data from a small region in Germany; however, the incidence figures were well comparable to a nationwide study 2005–2007 using statutory health insurance data [8].

The strengths of our study are that we could use a well-established population-based register and cover a long time span of 17 years. Furthermore, during the whole observation period 1998–2014 the method of case ascertainment, diagnostics definitions and techniques remained unchanged. Third, we were able to consider undetected diabetes in cases as well as in the background population.

### Conclusion

With regard to the objectives of the St. Vincent declaration, we found a substantial reduction in the IR of all stroke in the diabetic population which also tended do decrease for ischaemic stroke. In contrast, the IR did not materially change in the non-diabetic population with regard to all and ischaemic stroke This may indicate an improvement in diabetes care. However, future research in other populations is needed to confirm these findings.

## Supporting information

S1 Fig Age–sex standardised incidence rate of stroke with and without diabetes (per 100,000 person years): Assuming the diabetes prevalence to be constant up to 2011. Continuous lines = persons with diabetes; dotted lines = persons without diabetes; x-axis: calendar year; y-axis: incidence rate per 100,000 person years.
(TIF)

S2 Fig Age–sex standardised incidence rate of stroke with and without diabetes (per 100,000 person years): Assuming all first strokes with unknown diabetes status to be diabetic. Continuous lines = persons with diabetes; dotted lines = persons without diabetes; x-axis: calendar year; y-axis: incidence rate per 100,000 person years.
(TIF)

S3 Fig Age–sex standardised incidence rate of stroke with and without diabetes (per 100,000 person years): Assuming all first strokes with unknown diabetes status to be non-diabetic. Continuous lines = persons with diabetes; dotted lines = persons without diabetes; x-axis: calendar year; y-axis: incidence rate per 100,000 person years.
(TIF)

S1 Table Results of poisson models: Relative risks for stroke, Erlangen, 1998–2014: Assuming the diabetes prevalence to be constant up to 2011. (DOCX)

S2 Table Results of poisson models: Relative risks for stroke, Erlangen, 1998–2014: Assuming all first strokes with unknown diabetes status to be diabetic. (DOCX)

S3 Table Results of poisson models: Relative risks for stroke, Erlangen, 1998–2014: Assuming all first strokes with unknown diabetes status to be non-diabetic. (DOCX)
