# Supplementary figures and images for: Incidence and relative risk of stroke in the diabetic and the non-diabetic population between 1998 and 2014: A community-based stroke register
Source: PLoS One. 2017 Nov 16;12(11):e0188306. doi: 10.1371/journal.pone.0188306 (PMC5690660; doi:10.1371/journal.pone.0188306)

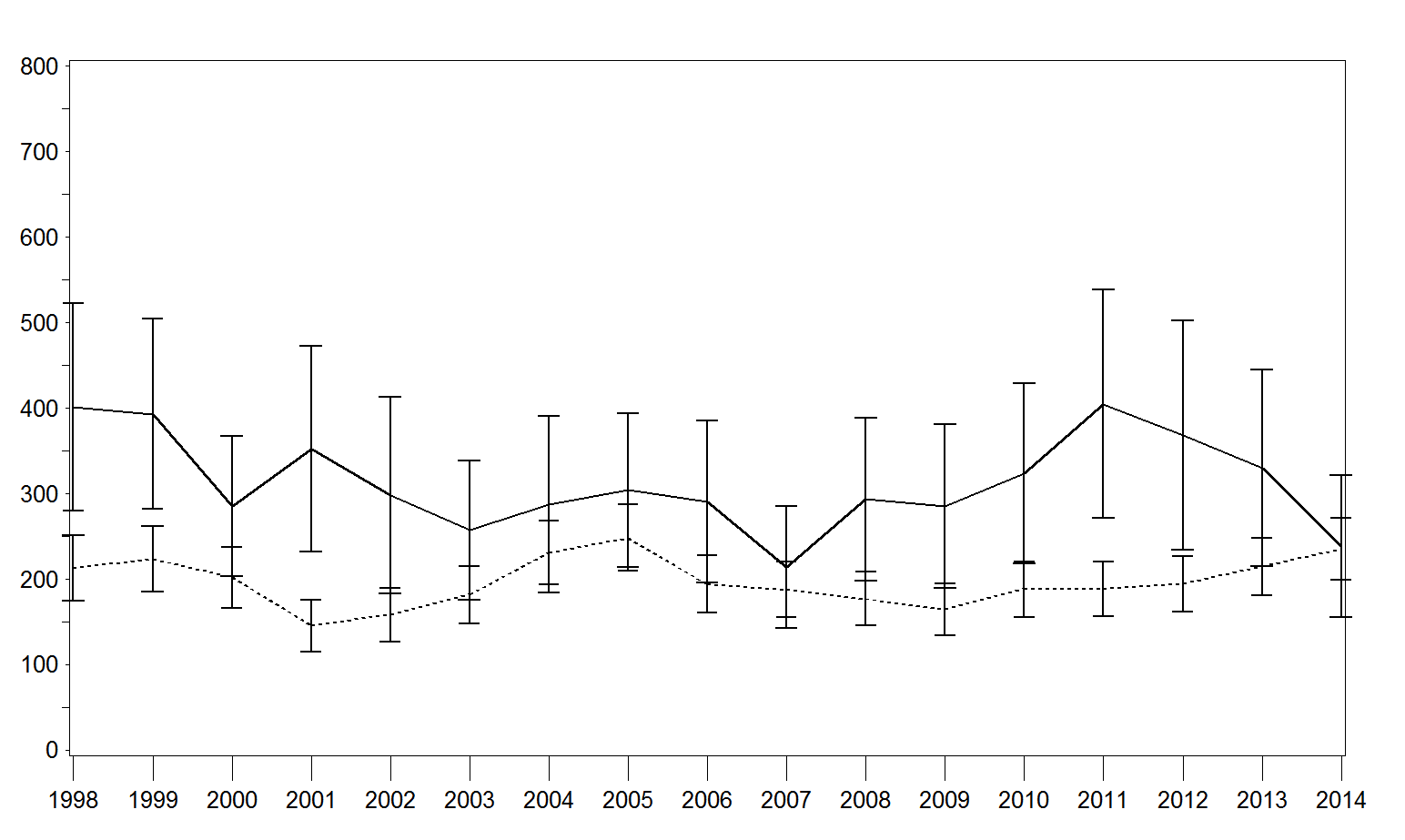

Supplement: S1 Fig — Continuous lines = persons with diabetes; dotted lines = persons without diabetes; x-axis: calendar year; y-axis: incidence rate per 100,000 person years. (TIF) [file pone.0188306.s001.tif]

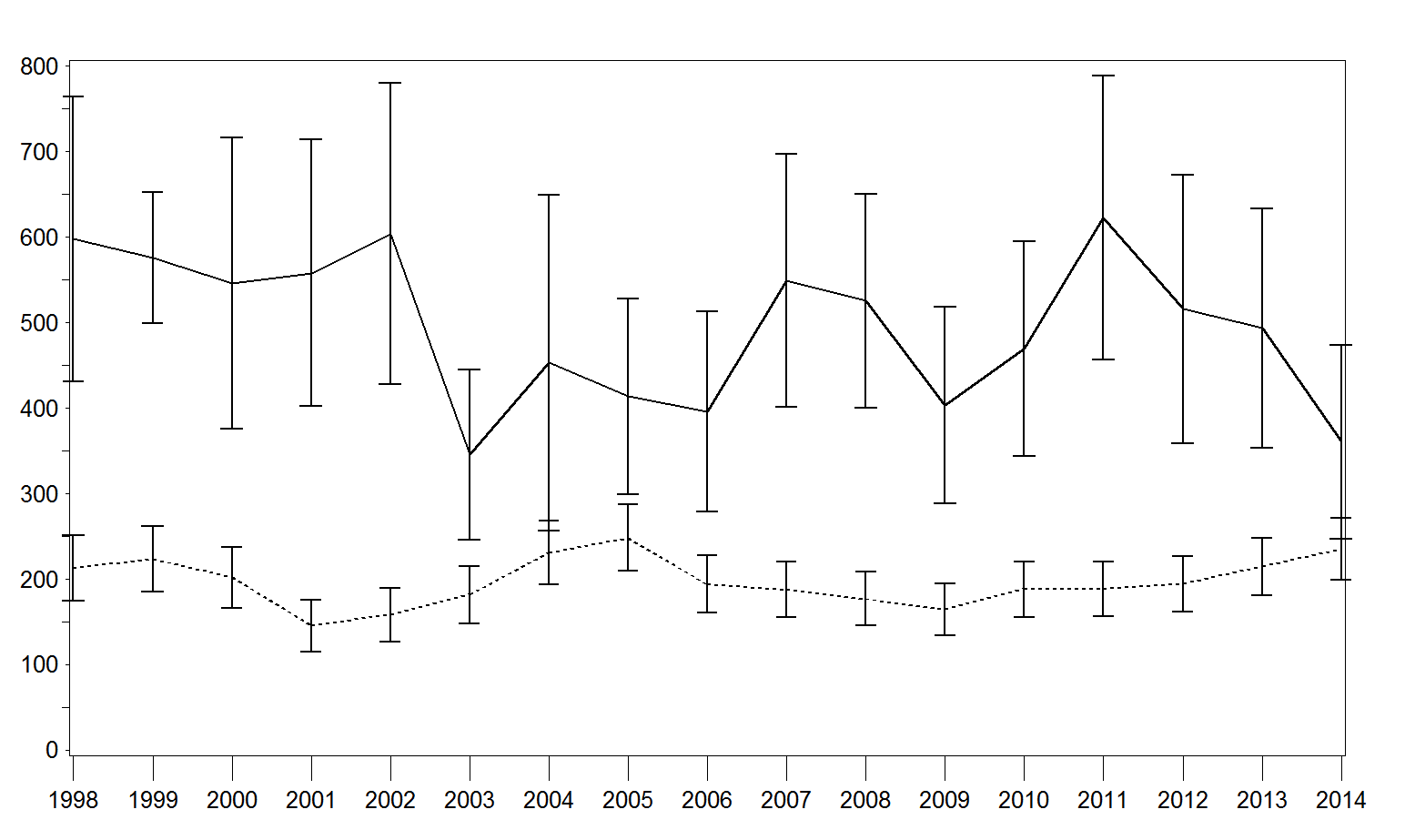

Supplement: S2 Fig — Continuous lines = persons with diabetes; dotted lines = persons without diabetes; x-axis: calendar year; y-axis: incidence rate per 100,000 person years. (TIF) [file pone.0188306.s002.tif]

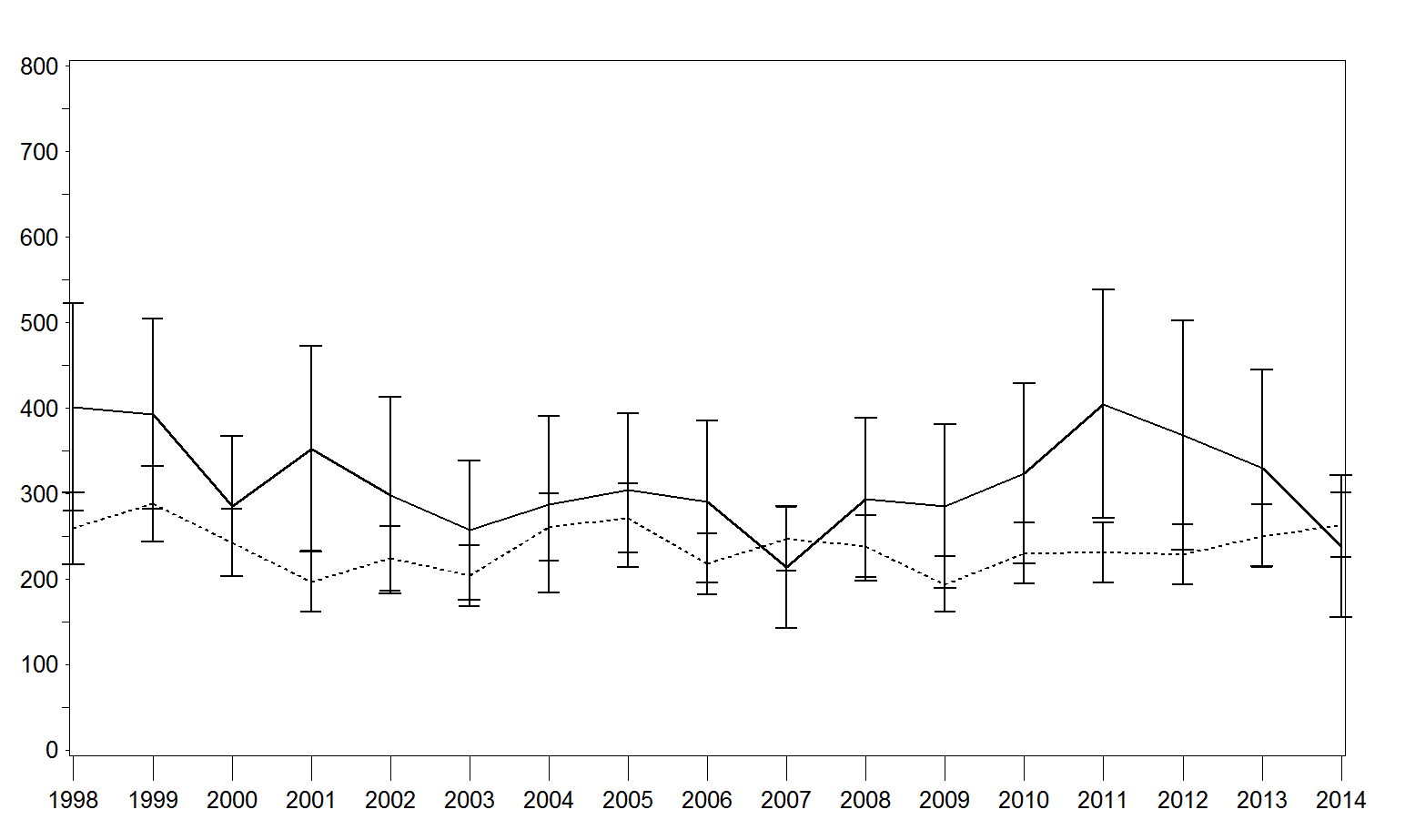

Supplement: S3 Fig — Continuous lines = persons with diabetes; dotted lines = persons without diabetes; x-axis: calendar year; y-axis: incidence rate per 100,000 person years. (TIF) [file pone.0188306.s003.tif]
