# Supplementary material for: Incidence and relative risk of stroke in the diabetic and the non-diabetic population between 1998 and 2014: A community-based stroke register
Source: PLoS One. 2017 Nov 16;12(11):e0188306. doi: 10.1371/journal.pone.0188306 (PMC5690660; doi:10.1371/journal.pone.0188306)
Supplement: S1 Table — (DOCX) [file pone.0188306.s004.docx]

| S1 Table: Results of Poisson models^a^: relative risks for Stroke, Erlangen, 1998-2014 | | | | | |
| --- | --- | --- | --- | --- | --- |
| Variables | Relative risk for Stroke (95% CI)^b^ | | |  | |
|  | Total population | Men | | Women | |
| Model 1a (diabetic) |  |  | |  | |
| Calendar year | 0.985 (0.972-0.999)** | | 0.988 (0.969-1.008) | | 0.985 (0.966-1.003) |
| Male vs. female | 1.265 (1.098-1.456)** | | --------- | | --------- |
| Age (years)* |  | |  | |  |
| ≥ 80 | 17.879 (11.157-28.653)** | | 9.900 (5.639-17.380)** | | 34.145 (15.222-76.596)** |
| 70-79 | 10.923 (6.823-17.487)** | | 7.947 (4.601-13.727)** | | 17.833 (7.921-40.150)** |
| 60-69 | 6.517 (4.020-10.565)** | | 5.33 (3.063-9.273)** | | 8.322 (3.582-19.333)** |
| 50-59 | 4.333 (2.568-7.313)** | | 3.053 (1.680-5.546)** | | 7.371 (2.937-18.500)** |
|  |  | |  | |  |
| Model 1b (non-diabetic) |  | |  | |  |
| Calendar year | 1.003 (0.993-1.013) | | 1.003 (0.991-1.015) | | 1.003 (0.989-1.017) |
| Male vs. female | 1.229 (1.113-1.357)** | | --------- | | --------- |
| Age (years) |  | |  | |  |
| ≥ 80 | 56.774 (46.857-68.790)** | | 56.430 (44.548-71.482)** | | 51.852 (39.931-67.332)** |
| 70-79 | 28.067 (23.109-34.089)** | | 33.473 (26.617-42.094)** | | 23.202 (17.678-30.453)** |
| 60-69 | 12.651 (10.285-15.561)** | | 17.195 (13.559-21.806)** | | 8.938 (6.619-12.069)** |
| 50-59 | 5.468 (4.344-6.884)** | | 7.959 (6.168-10.270)** | | 3.354 (2.346-4.793)** |
|  |  | |  | |  |
| Model 2 |  | |  | |  |
| Calendar year | 1.004 (0.994-1.014) | | 1.004 (0.991-1.018) | | 1.004 (0.990-1.017) |
| Diabetes (yes vs. no) | 1.725 (1.440-2.061)** | | 1.874 (1.468-2.380)** | | 1.566 (1.233-1.979)** |
| Male vs. female | 1.252 (1.151-1.361)** | | --------- | | --------- |
| Age (years) |  | |  | |  |
| ≥ 80 | 49.497 (41.474-59.483)** | | 44.795 (35.291-57.431)** | | 48.984 (38.813-62.686)** |
| 70-79 | 26.086 (21.839-31.371)** | | 29.152 (23.173-37.088)** | | 22.973 (18.074-29.579)** |
| 60-69 | 12.723 (10.549-15.433)** | | 16.320 (12.892-20.873)** | | 9.206 (7.063-12.106)** |
| 50-59 | 5.774 (4.680-7.144)** | | 7.731 (5.986-10.058)** | | 3.767 (2.739-5.177)** |
| Diabetes x calendar year | 0.979 (0.961-0.997)** | | 0.979 (0.955-1.003) | | 0.980 (0.955-1.004) |
|  |  |  | |  | |

^a^models were adjusted for all variables included in this table

^b^baseline: 18-49 years

^c^ P<.05
