# Supplementary material for: Incidence and relative risk of stroke in the diabetic and the non-diabetic population between 1998 and 2014: A community-based stroke register
Source: PLoS One. 2017 Nov 16;12(11):e0188306. doi: 10.1371/journal.pone.0188306 (PMC5690660; doi:10.1371/journal.pone.0188306)
Supplement: S2 Table — (DOCX) [file pone.0188306.s005.docx]

| S2 Table: Results of Poisson models^a^: relative risks for Stroke, Erlangen, 1998-2014 | | | | | |  |
| --- | --- | --- | --- | --- | --- | --- |
| Variables | Relative risk for Stroke (95% CI)^b^ | | |  | |  |
|  | Total population | Men | | Women | |  |
| Model 1a (diabetic) |  |  | | |  |  |
| Calendar year | 0.982 (0.970-0.995)** | 0.977 (0.959-0.995)** | | | 0.988 (0.972-1.005) | |
| Male vs. female | 1.114 (0.981-1.266) | --------- | | | --------- | |
| Age (years)* |  |  | | |  | |
| ≥ 80 | 14.246 (9.965-20.366)** | | 8.818 (5.653-13.754)** | | 22.997 (12.966-40.788)** | |
| 70-79 | 6.981 (4.872-10.004)** | | 5.229 (3.378-8.093)** | | 10.154 (5.687-18.130)** | |
| 60-69 | 3.863 (2.649-5.632)** | | 3.168 (2.023-4.962)** | | 4.718 (2.541-8.760)** | |
| 50-59 | 3.130 (2.069-4.736)** | | 2.219 (1.363-3.613)** | | 4.880 (2.444-9.741)** | |
|  |  | |  | |  | |
| Model 1b (non-diabetic) |  | |  | |  | |
| Calendar year | 1.003 (0.993-1.013) | | 1.003 (0.991-1.016) | | 1.003 (0.989-1.017) | |
| Male vs. female | 1.233 (1.117-1.361)** | | --------- | | --------- | |
| Age (years) |  | |  | |  | |
| ≥ 80 | 56.831 (46.908-68.853)** | | 56.607 (44.655-71.758)** | | 51.784 (39.893-67.218)** | |
| 70-79 | 28.096 (23.134-34.121)** | | 33.575 (26.680-42.252)** | | 23.170 (17.660-30.399)** | |
| 60-69 | 12.637 (10.274-15.542)** | | 17.187 (13.542-21.812)** | | 8.918 (6.607-12.037)** | |
| 50-59 | 5.462 (4.340-6.875)** | | 7.947 (6.154-10.263)** | | 3.350 (2.345-4.786)** | |
|  |  | |  | |  | |
| Model 2 |  | |  | |  | |
| Calendar year | 1.004 (0.993-1.014) | | 1.004 (0.989-1.019) | | 1.003 (0.989-1.018) | |
| Diabetes (yes vs. no) | 2.716 (2.294-3.211)** | | 2.882 (2.273-3.645)** | | 2.513 (2.013-3.131)** | |
| Male vs. female | 1.196 (1.099-1.300)** | | --------- | | --------- | |
| Age (years) |  | |  | |  | |
| ≥ 80 | 46.119 (38.54-55.588)** | | 42.147 (32.955-54.49)** | | 46.261 (36.358-59.756)** | |
| 70-79 | 22.687 (18.923-27.391)** | | 24.979 (19.664-32.109)** | | 20.581 (16.05-26.763)** | |
| 60-69 | 11.054 (9.122-13.471)** | | 13.713 (10.714-17.74)** | | 8.480 (6.442-11.272)** | |
| 50-59 | 5.513 (4.451-6.848)** | | 7.102 (5.441-9.339)** | | 3.826 (2.756-5.310)** | |
| Diabetes x calendar year | 0.977 (0.96-0.994)** | 0.969 (0.946-0.993)** | | | 0.984 (0.962-1.007) | |
| ^a^models were adjusted for all variables included in this table  ^b^Baseline: 18-49 years  ^c^ P<.05 | | | | | |  |
