# Supplementary material for: Incidence and relative risk of stroke in the diabetic and the non-diabetic population between 1998 and 2014: A community-based stroke register
Source: PLoS One. 2017 Nov 16;12(11):e0188306. doi: 10.1371/journal.pone.0188306 (PMC5690660; doi:10.1371/journal.pone.0188306)
Supplement: S3 Table — (DOCX) [file pone.0188306.s006.docx]

| S3 Table: Results of Poisson models^a^: relative risks for Stroke, Erlangen, 1998-2014 | | | | |
| --- | --- | --- | --- | --- |
| Variables | Relative risk for Stroke (95% CI)^b^ | | |  |
|  | Total population | Men | | Women |
| Model 1a (diabetic) |  |  | |  |
| Calendar year | 0.985 (0.972-0.999)** | | 0.987 (0.968-1.007) | 0.985 (0.967-1.004) |
| Male vs. female | 1.254 (1.089-1.443)** | | --------- | --------- |
| Age (years)* |  | |  |  |
| ≥ 80 | 17.963 (11.209-28.787)** | | 9.847 (5.620-17.254)** | 34.681 (15.436-77.92)** |
| 70-79 | 10.976 (6.856-17.573)** | | 7.907 (4.586-13.631)** | 18.118 (8.034-40.857)** |
| 60-69 | 6.600 (4.071-10.701)** | | 5.348 (3.080-9.288)** | 8.509 (3.657-19.803)** |
| 50-59 | 4.410 (2.613-7.443)** | | 3.084 (1.701-5.591)** | 7.528 (2.994-18.929)** |
|  |  | |  |  |
| Model 1b (non-diabetic) |  | |  |  |
| Calendar year | 0.998 (0.989-1.007) | | 0.998 (0.986-1.010) | 0.999 (0.988-1.011) |
| Male vs. female | 1.187 (1.084-1.298)** | | --------- | --------- |
| Age (years) |  | |  |  |
| ≥ 80 | 65.152 (54.551-77.813)** | | 63.259 (50.190-79.732)** | 61.446 (48.850-77.290)** |
| 70-79 | 29.651 (24.741-35.536)** | | 33.563 (26.737-42.131)** | 25.869 (20.370-32.851)** |
| 60-69 | 12.423 (10.228-15.088)** | | 16.231 (12.800-20.582)** | 9.207 (7.061-12.006)** |
| 50-59 | 5.543 (4.471-6.872)** | | 7.814 (6.063-10.071)** | 3.536 (2.586-4.835)** |
|  |  | |  |  |
| Model 2 |  | |  |  |
| Calendar year | 0.999 (0.990-1.008) | | 0.999 (0.986-1.013) | 0.999 (0.988-1.011) |
| Diabetes (yes vs. no) | 1.358 (1.134-1.621)** | | 1.542 (1.199-1.971)** | 1.197 (0.953-1.496) |
| Male vs. female | 1.215 (1.121-1.316)** | | --------- | --------- |
| Age (years) |  | |  |  |
| ≥ 80 | 56.877 (47.990-67.860)** | | 50.449 (39.917-64.408)** | 57.730 (46.485-72.596)** |
| 70-79 | 27.931 (23.525-33.378)** | | 29.884 (23.799-37.939)** | 25.685 (20.537-32.497)** |
| 60-69 | 12.784 (10.653-15.421)** | | 15.921 (12.584-20.344)** | 9.587 (7.481-12.385)** |
| 50-59 | 5.856 (4.776-7.198)** | | 7.691 (5.961-9.991)** | 3.914 (2.911-5.263)** |
| Diabetes x calendar year | 0.982 (0.964-1.001) | | 0.981 (0.956-1.006) | 0.984 (0.960-1.008) |

^a^models were adjusted for all variables included in this table

| ^b^Baseline: 18-49 years |
| --- |
| ^c^ P<.05 |
